# Natural Products, Traditional Uses and Pharmacological Activities of the Genus *Biebersteinia* (Biebersteiniaceae)

**DOI:** 10.3390/plants9050595

**Published:** 2020-05-07

**Authors:** Benyin Zhang, Xiaona Jin, Hengxia Yin, Dejun Zhang, Huakun Zhou, Xiaofeng Zhang, Lam-Son Phan Tran

**Affiliations:** 1 State Key Laboratory of Plateau Ecology and Agriculture, Qinghai University, Xining 810016, China; hengxiayin@qhu.edu.cn (H.Y.); djzhang@nwipb.cas.cn (D.Z.); 2 Laboratory of Natural Product Research, College of Eco-Environmental Engineering, Qinghai University, Xining 810016, China; 3 Institute of Life Science Application, College of Medicine, Xi’an International University, Xi’an 710017, China; xaiu05029@xaiu.edu.cn (X.J.); xaiu16041@xaiu.edu.cn (X.Z.); 4 The Key Laboratory of Restoration Ecology in Cold Region of Qinghai Province, Northwest Institute of Plateau Biology, Chinese Academy of Science, Xining 810008, China; hkzhou@nwipb.cas.cn; 5 Institute of Research and Development, Duy Tan University, 03 Quang Trung, Da Nang 550000, Vietnam; 6 Stress Adaptation Research Unit, RIKEN Center for Sustainable Resource Science, 1-7-22, Suehiro-cho, Tsurumi, Yokohama 230-0045, Japan

**Keywords:** *Biebersteinia*, Biebersteiniaceae, chemical properties, natural products, traditional uses, pharmacological activities

## Abstract

Medicinal plants have been known as a rich source of natural products (NPs). Due to their diverse chemical structures and remarkable pharmacological activities, NPs are regarded as important repertoires for drug discovery and development. *Biebersteinia* plant species belong to the Biebersteiniaceae family, and have been used in folk medicines in China and Iran for ages. However, the chemical properties, bioactivities and modes of action of the NPs produced by medicinal *Biebersteinia* species are poorly understood despite the fact that there are only four known *Biebersteinia* species worldwide. Here, we reviewed the chemical classifications and diversity of the various NPs found in the four known *Biebersteinia* species. We found that the major chemical categories in these plants include flavonoids, alkaloids, phenylpropanoids, terpenoids, essential oils and fatty acids. We also discussed the anti-inflammatory, analgesic, antibacterial, antioxidant, antihypertensive and hypoglycemic effects of the four *Biebersteinia* species. We believe that the present review will facilitate the exploration of traditional uses and pharmacological properties of *Biebersteinia* species, extraction of the NPs and elucidation of their molecular mechanisms, as well as the development of novel drugs based on the reported properties and mode-of-action.

## 1. Introduction

*Biebersteinia* is the smallest genus of Biebersteiniaceae. The systemic and taxonomic position of this genus has long been in dispute due to its rare species and limited availability of representative herbarium collections [1]. The genus was traditionally positioned in Geraniales 30 years ago, but now it was accepted that it belongs to Sapindales as a separate order and family based on the molecular phylogenetic analysis [2,3]. The genus *Biebersteinia* was originally recognized to comprise five species—namely, *B. heterostemon* Maxim., *B. multifida* DC., *B. leiosepala* Jaub. & Spach, *B. odora* Stephan ex Fisch. and *B. orphanidis* Boiss. decades ago [1]. However, *B. leiosepala* is now recognized to be a synonymous species of *B. multifida* (http://www.theplantlist.org/, http://www.worldfloraonline.org/, and https://www.gbif.org/); and thus, there are a total of four species in the genus. These species are widely distributed in mountainous, semi-arid regions from central and western Asia to the eastern Mediterranean [1,2,4,5,6].

All of the *Biebersteinia* species are perennial herbs, and possess slightly different biological characteristics and geographical distributions. *B. heterostemon*, also called “Xun Dao Niu” in Chinese, is endemic to the Qinghai-Tibetan Plateau and its adjacent regions in China [6]. This plant species inhabits arid and semi-arid alpine deserts, rocky slopes and other environments (http://www.iplant.cn/foc/). The morphological and biological characteristics of *B. heterostemon* are 40–120 cm tall, lanceolate leaf blade bearing long simple hairs and small stipitate glands, flowers in two or three fascicles with hairy or glandular pedicel, as well as yellow and obovate petals (http://www.iplant.cn/foc/). *B. multifida* is a common herb known as Adamak in Iran, with 20–70 cm long stem, laciniate leaves, flowers formed in a lax panicle, calyx strengthened in fruit, and yellowish petals slightly shorter than the sepals [7]. *B. odora* is distributed widely across central Asia (e.g., Kazakhstan, Kyrgyzstan, Pakistan, India, China and Mongolia) and inhabited in alpine meadows and dry rocky and scree slopes. *B. odora* is 10–30 cm tall, and has pinnatisect leaves and yellow flowers with orange center (1–1.5 cm across, in racemes) [8]. *B. orphanidis* is the only species distributed in Europe and found in Greece besides Asia. *B. orphanidis* grows at altitudes ~1400–1750 m in deep sandy-clay soil in dolines over limestone, usually in openings of *Abies cephalonica* forest. These plants are 15–40 cm long, broadly oblanceolate in outline, with scarious stipules and short petioles [5].

Of these species, *B. heterostemon* and *B. multifida* have long histories as traditional folk medicines in Iran and the Tibetan region of China, respectively, and have been used to treat various diseases. Modern pharmacological studies have shown that these two plant species have significant pharmaceutical effects on humans, and possess various ethnomedicinal properties, including antioxidant, analgesic, anti-inflammatory, antispasmodic, hypoglycemic, hypotensive and anti-atherosclerotic properties [9,10,11]. Therefore, numerous phytochemists and pharmacologists worldwide have investigated the pharmacodynamically active substances in *Biebersteinia* species. Natural products isolated from *Biebersteinia* plants include flavonoids, guanidines, alkaloids, phenylpropanoids, terpenoids, sterols and fatty acids, as well as various compounds of essential oils. The present review summarizes the findings of several decades of research into the chemical constituents and pharmacological functions of the four identified *Biebersteinia* species. This review, therefore, will facilitate further investigations into the complete chemical profile of the secondary metabolites in these plants, as well as their pharmacological activities and molecular mechanisms.

## 2. Data Collections

All data presented in this review were summarized from the references, including scientific journals, book chapters or dissertations. These references were systematically searched against electronic databases: PubMed, CNKI (http://new.oversea.cnki.net/index/), Web of Science, Scopus and Google Scholar with a keyword “*Biebersteinia*”. To search for maximum relative references, the keyword was set as “*Biebersteinia”* without any other restrictions. Subsequently, references closely related to chemical compositions, traditional uses and pharmacological properties, including in vitro and in vivo investigations, were screened for further data extraction. In addition, to survey the taxon, phenotypes and geographical distributions of species in *Biebersteinia*, several online taxonomic databases, including http://theplantlist.org/, http://www.worldfloraonline.org/, https://www.gbif.org/ and http://www.iplant.cn/foc/, were also explored.

## 3. Natural Products Isolated from Bieberstrinia

### 3.1. Flavonoids

**Figure 1:**
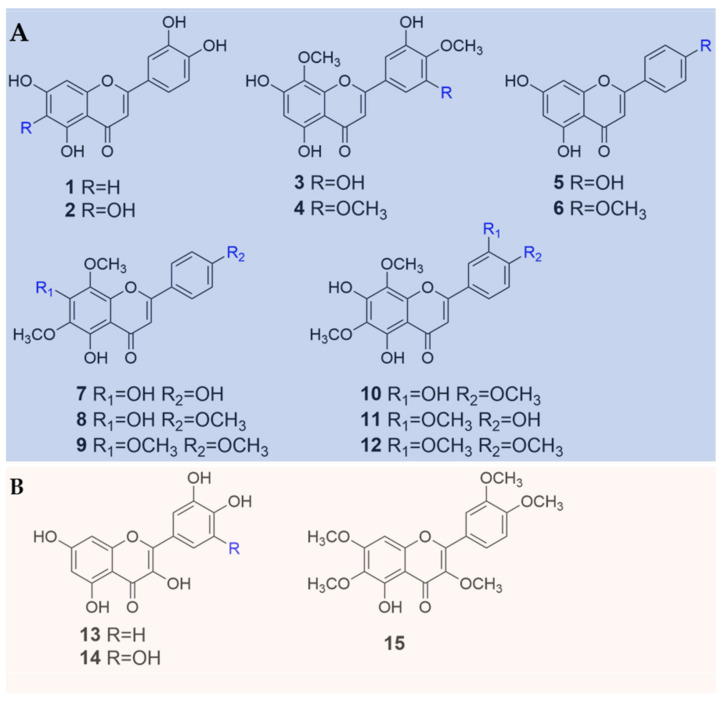
Chemical structures of 15 flavonoid aglycones identified in *Biebersteinia* plants. (**A**) flavone aglycones; (**B**) flavonol aglycones.

**Figure 2:**
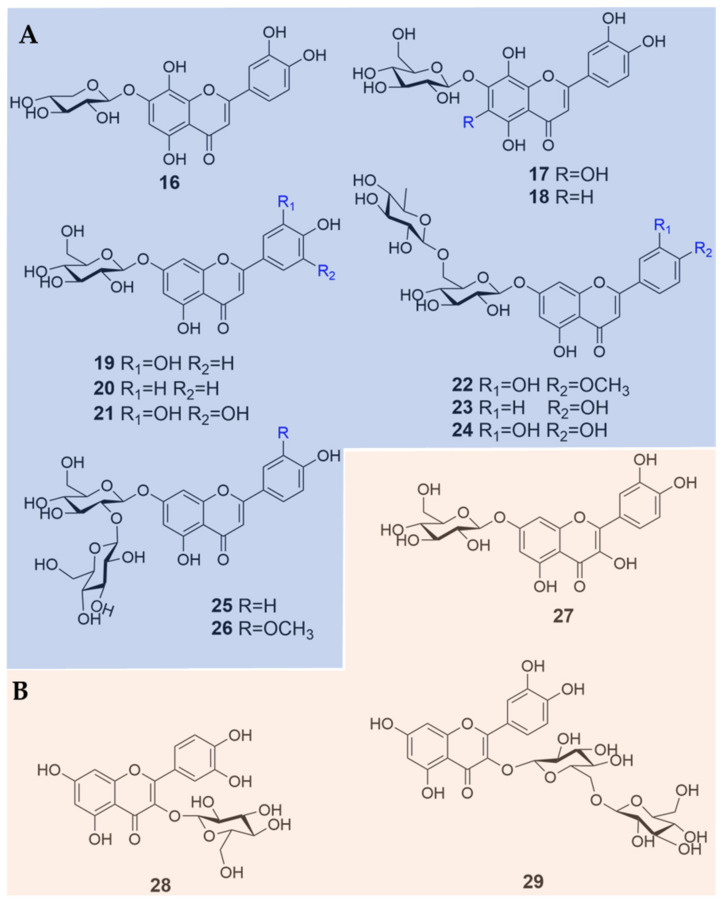
Chemical structures of 14 flavonoid glycosides identified in *Biebersteinia* plants. (**A**) flavone glycosides; (**B**) flavonol glycosides.

Up-to-date, 29 flavonoids (Figure 1 and Figure 2; Table 1) have been isolated from four *Biebersteinia* species, which occupies most of the known chemicals in *Biebersteinia* species. The flavonoid aglycones comprise mainly flavones or flavonols, such as quercetin, luteolin and apigenin. Fifteen aglycone derivatives have been discovered, among which 12 compounds are flavones (**1**–**12**) and three are flavonols (**13**–**15**) (Figure 1; Table 1). Fourteen flavonoid glycosides with different types or numbers of glycosyl moiety, including 11 flavone (**16**–**26**) and three flavonol glycosides (**27**–**29**) were found (Figure 2; Table 1).

In addition, most of the flavonoid aglycones and glycosides were highly hydroxy- or methoxy-substituted at C-6, C-8, C-3’, C-4’ and C-5’ in their chemical structures (Figure 1 and Figure 2). Both C-6 and C-8 were substituted by methoxy groups as seen in the chemical structures of compounds **7**–**12** (Figure 1). To the best of our knowledge, this configuration occurs rarely in nature, which might be correlated with their extreme habitats. From the sources of flavonoids, 18 compounds were isolated from the species *B. heterostemon* (**1**–**4**, **13**, **15**, **16**–**19** and **22**–**29**) [11,12,13,14], among which compounds **3** and **18** were recently discovered by our group from the species for the first time [14]. Compounds **1**, **19**, **23**, **5**–**12** and **20**–**21** were mainly identified from *B. orphanidis* [15], while three flavonoids (**1**, **7** and **12**) were found in *B. multifida*; however, only one compound, namely myricetin (**14**), was reported from *B. odora* [16] (Table 1).

The content of total flavonoids (CTF) in plants may be correlated with their habitats, ecological roles and responses to abiotic and/or biotic stresses [17,18,19]. In general, the *Biebersteinia* species are widely distributed at high elevations, and are exposed to extreme drought, low temperatures and strong ultraviolet radiation [20]. All of these conditions could induce high production of CTF. One of our previous studies showed that the CTF reached 0.24% in *B. heterostemon* located on the Qinghai-Tibetan Plateau [21]. CTF may also widely vary among different plant organs and tissues. For instance, in *B. multifida*, leaves were found to have the highest CTF (39.9 ± 2.1 mg/g), followed by flowers, stems and roots [22].

### 3.2. Guanidines

**Figure 3:**
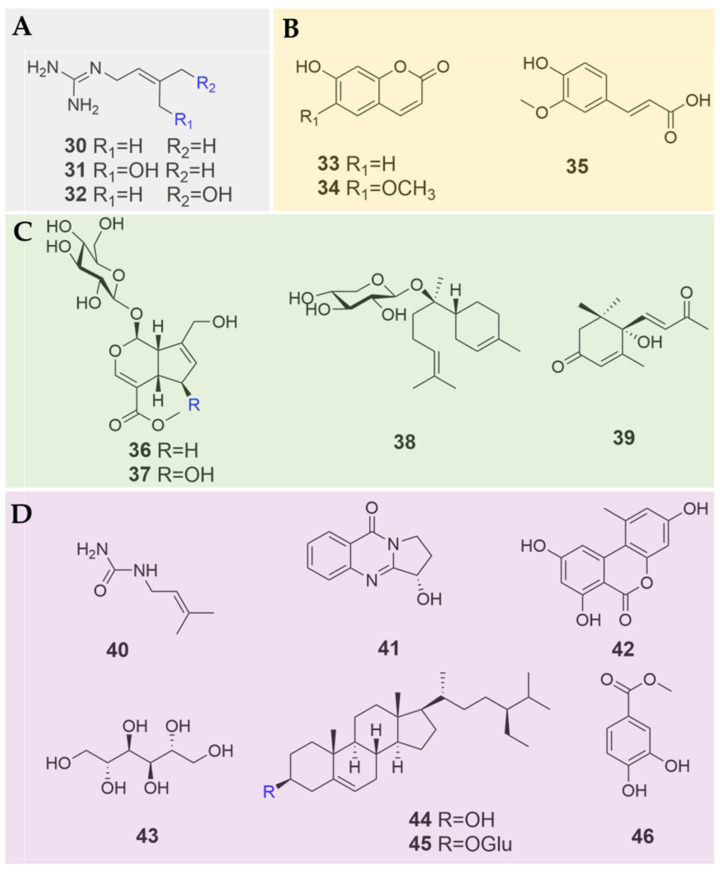
Chemical structures of guanidines (**A**) phenylpropanoids, (**B**) terpenoids, (**C**) and other compounds, (**D**) identified in *Biebersteinia* species.

Three rare prenylated guanidines, namely galegine (**30**), *cis*-4-hydroxy galegine (**31**) and *trans*-4-hydroxy galegine (**32**) [30,31], have been found in *B. heterostemon* [25] (Figure 3A; Table 1). The clinical hypoglycemic drug metformin was derived from galegine, which might account for the hypoglycemic efficacy of the traditional galegine-containing Tibetan medicine *B. heterostemon.* In fact, numerous alkaloids [14], such as coptisonine [32], conophylline [33] and vindogentianine [34], can induce hypoglycemia.

### 3.3. Phenylpropanoids

Three phenylpropanoids have been isolated so far from two *Biebersteinia* species (Figure 3B; Table 1). Compounds **33** and **34** are coumarins that were identified in *B. multifida*, which differ only in terms of their substituents at C-6. Compound **35** is a ferulic acid that was isolated from *B. multifida* [23], as well as from *B. heterostemon* by our group (unpublished data).

### 3.4. Terpenoids

Until the present, four terpenoids have been isolated from *B. heterostemon* among the four *Biebersteinia* species. These identified terpenoids include two iridoid glucosides, i.e., geniposide (**36**) and 6β-hydroxygeniposide (**37**), and one sesquiterpene glycoside (-)-anymol-8-*O*-β-D-lyxopyranoside (**38**) [26]. They are the main active ingredients [35], and are easily hydrolyzed by β-glucosidase to genipin [36]. In addition, we recently isolated one sesquiterpene (+)-dehydrovomifoliol (**39**) from *B. heterostemon*, the identification and characterization of which were also reported for the first time from the genus *Biebersteinia* (Figure 3C; Table 1).

### 3.5. Other Compounds

Seven other types of compounds were isolated from *B. heterostemon*, including *N*-3-methyl-2-butenylurea (**40**) [11], alkaloid vasicinone (**41**) [27], alternariol (**42**) [24], mannitol (**43**) [12], β-sitosterol (**44**) [11,12,24], daucosterol (**45**) [12], and protocatechuic acid methyl ester (**46**) [24] (Figure 3D; Table 1). In addition, three neutral polysaccharides were obtained from the roots of *B. multifida*, namely, glucan-A, glucan-B and glucan-C. Their molecular weights were 4100, 2200 and 1100, respectively [37,38,39,40].

### 3.6. Fatty Acids

**Figure 4:**
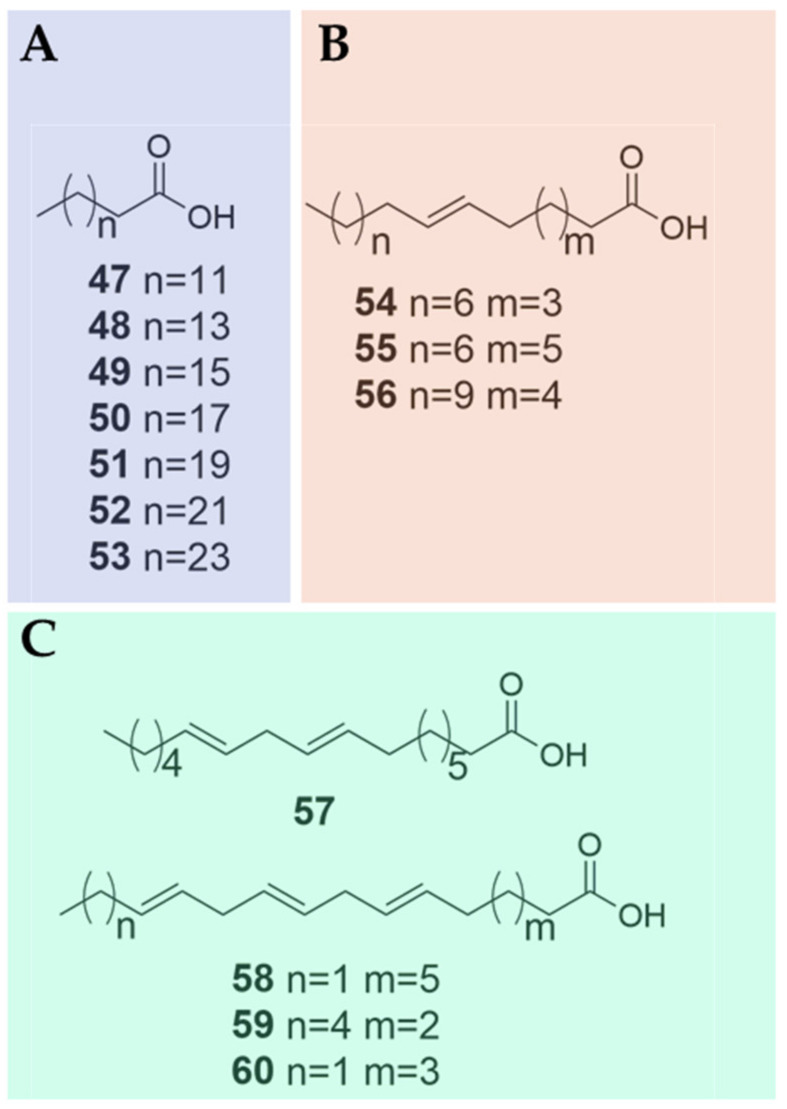
Chemical structures of fatty acids identified in *Biebersteinia* species. (**A**) saturated fatty acids; (**B**) monounsaturated fatty acids; (**C**) polyunsaturated fatty acids.

Fatty acids, which are aliphatic monocarboxylic acids, can either be saturated or unsaturated depending on the absence or presence of double bonds [41]. A number of studies have reported the presence of various fatty acids in two out of four *Biebersteinia* species [28,29]. In particular, a total of 14 fatty acids were identified in the seed oil of *B. heterostemon* and leaves of *B. orphanidis* (Figure 4; Table 1). These fatty acids include seven saturated fatty acids (**47**–**53**), three monounsaturated fatty acids (**54**–**56**), and four polyunsaturated fatty acids (**57**–**60**). By using gas chromatography (GC) analysis of fatty acids in seed oil of *B. heterostemon*, nine fatty acids (**48**–**50** and **54**–**59**) were identified, which accounted for 88.44% of total fatty acid content that mainly consisted of unsaturated fatty acids, such as oleic (**55**), linoleic (**57**) and linolenic (**58** and **59**) acids, while the lower detected part (7.94%) of total fatty acid content contained saturated fatty acids, mainly palmitic (**48**) and stearic (**49**) acids [29]. In particular, the content of linoleic acid reached 73.04% [29]. Twelve fatty acids were elucidated in the leaves of *B. orphanidis* (**47**–**49**, **51**–**58** and **60**), among which palmitic, linolenic and linoleic acids were predominant, representing 30.60%, 21.83% and 11.67%, respectively, in total fatty acid content [28].

## 4. Chemical Compositions of Essential Oils in Biebersteinia Species

Up-to-date, 112 chemical constituents have been identified in the essential oils of three *Biebersteinia* species, namely *B. multifida*, *B. heterostemon* and *B. orphanidis* [42,43,44,45,46,47,48], mainly by using gas chromatography-mass spectrometry (GC-MS) analyses (Table 2). In particular, the chemical compositions of essential oils of *B. multifida* were more systematically investigated, using different types of tissues, such as leaves, fruits and roots [45], or using different extraction methods, such as hydrodistillation, microwave, solvent and supercritical fluid extraction (SFE) [42,44]. In the essential oil of *B. multifida*, a total of 88 chemical constituents were identified [45,46,47,48]. The chemodiversity and contents of various compounds in essential oils from different parts of *B. multifida* differed significantly [45]. Specifically, thymol (16.5% of total essential oil), α-pinene (14.3%), β-pinene (12.4%), β-caryophyllene (11.2%) and 1,8-cineol (10.1%) are the major compounds in essential oil extracted from *B. multifida* leaves; thymol (38.4%), 1,8-cineol (18.4%), γ-terpinene (11.3%) and β-caryophyllene (9.8%) are the main compounds in essential oil extracted from *B. multifida* roots; and thymol (30.9%), β-caryophyllene (15.5%), α-pinene (9.4%), β-pinene (8.8%), caryophyllene oxide (8.4%) and limonene (7.5%) are predominant in essential oil extracted from *B. multifida* fruits [45]. Additionally, different extraction methods were also shown to induce different chemical types or contents in essential oil extracts of *B. multifida*. For example, the hydrodistillation method enabled the authors to mainly detect (*E*)-nerolidol (31.45%) and phytol (17.1%); microwave extraction allowed detection of (*E*)-nerolidol (28.4%), *n*-heptacosane (17.36%), *n*-docosane (12.97%) and 6,10,14-trimethyl-2-pentadecanone (10.38%); while solvent extraction detected mainly nonacosane (38.62%), mandenol (17.17%) and *n*-heptacosane (10.23%) [44]. In addition to the above-mentioned extraction approaches of essential oils, the supercritical fluid extraction (SFE) is a green technology that has been widely used in the past few decades to extract essential oils, nonpolar substances, fatty acids, phytosterols, and other functional and nutraceutical components from natural sources [49,50,51,52,53,54]. Four compounds, namely nerolidol, 6,10,14-trimethyl-2-pentadecanone, hexadecanoic acid and phytol that possess strong antioxidant activities, were the major components in essential oils extracted from aerial parts of *B. multifida* by both hydrodistillation and SFE methods; however, the yield of these four compounds extracted by SFE (91.74%) was far higher than that by the hydrodistillation method [42].

In comparison with *B. multifida*, the investigations of chemical compositions of essential oils extracted from *B. heterostemon* and *B. orphanidis* were still limited. Forty compounds (82.43% of total essential oil) were identified in the essential oil of aerial parts of *B. heterostemon*, mainly containing β-caryophyllene (33.79%), elixene (5.09%), β-elemenone (4.45%), germacrene D (3.64%), camphor (3.34%), α-bisabolol (3.30%) and geraniol (3.27%) [48]. Thirteen components constituting 98.12% of the essential oil extracted from the aerial parts of *B. orphanidis* were detected, and the major chemical constituents included *cis*-limonene oxide (47.90%), β-caryophyllene (9.70%) and α-bisabolol (8.23%) [43]. Furthermore, oxygenated monoterpenes (51.25%) were found to be predominated over other chemical types in the constituent composition of the *B. orphanidis* essential oil in this study [43].

## 5. Applications in Traditional Medicines

Among the four well-known *Biebersteinia* spp., only *B. heterostemon* and *B. multifida* have been commonly applied as traditional herbal medicines to treat musculoskeletal disorders, bone fractures and skin diseases [7,25,55]. In China, *B. heterostemon* plants are widely distributed in Qinghai-Tibetan Plateau, and have been administered as traditional Tibetan medicines [20,56]. In addition, *B. multifida* is indigenous to Iran, where this plant species has been topically applied as a folk remedy for treatments of muscle and skeletal disorders and bone fractures [9,57]. Besides, it has also been reported that children’s nocturia can be treated with *B. multifida* [58]. In addition, *B. odora* has been used in treatments of migraine and fever for centuries by people living in the Shigar Valley, Baltistan region of Karakorum range, Pakistan [8]. As *Biebersteinia* species have high pharmacological values as traditional medicines, their bioactivities have attracted the attention of a large number of phytochemists and pharmacologists.

## 6. Pharmacological Activities

### 6.1. In Vivo Pharmacological Activities

#### 6.1.1. Anti-Inflammatory and Analgesic Effects

The anti-inflammatory effect of *B. heterostemon* has been evaluated with a xylene-induced murine inflammation model. Its analgesic effect on mice was established by the hotplate and tail flick methods and by acetic acid-induced writhing [55]. Traditional *B. heterostemon* decoctions, traditional *B. heterostemon* decoctions followed by alcohol precipitation, and ethanolic *B. heterostemon* extracts inhibited xylene-induced ear edema in mice and elevated the mouse hotplate pain threshold [55]. However, the anti-inflammatory and analgesic efficacies of the ethanolic *B. heterostemon* extract were significantly stronger than those of the other afore-mentioned extracts [55]. These results might be correlated with those for *N*-3-methyl-2-butenyl urea (**40**) isolated from the ethanolic extract of *B. heterostemon*, as this compound was confirmed to have analgesic activity [11]. Similar findings were obtained and reported for *B. multifida*. A dose of 10 mg/kg *B. multifida* root extract obtained by ethanol refluxing, and that of 4 mg/kg indomethacin had similar anti-inflammatory efficacies in a carrageenan-induced edema assay [57]. The first phase of a formalin test indicated that the analgesic efficacy of 50 mg/kg *B. multifida* root extract was comparable to that of 10 mg/kg morphine [57]. These findings collectively indicate the high potential of *B. heterostemon* and *B. multifida* for the production of anti-inflammatory and analgesic drugs.

#### 6.1.2. Anti-Hypertensive and Hypoglycemic Effects

The compound *N*-3-methyl-2-butenyl urea (**40**) isolated from *B. heterostemon* displayed both analgesic and antihypertensive activities [11]. Numerous alkaloids from natural resources exhibited hypoglycemic effects [14]. *B. heterostemon* alkaloids showed significant hypoglycemic efficacy in streptozotocin-induced diabetic mice, with the optimum therapeutic dose at 5 mg/kg [59]. On the other hand, neither antihypertensive nor hypoglycemic activity was detected in any other *Biebersteinia* species. In addition, galegine (**30**), an isopentenyl guanidine, which was originally isolated from *Galega officinalis* and has significant hypoglycemic activity [60], was also detected in *B. heterostemon* [25]. In fact, the hypoglycemic drug metformin is a derivative of galegine [61,62,63,64].

#### 6.1.3. Anti-Fatigue and Anxiolytic Effects

The anti-fatigue effect of *B. multifida* root extract was also validated in a forced swimming test (FST), and the biochemical parameters in the blood related to fatigue were measured [7]. The results demonstrated the potential benefit of *B. multifida* root extract as an anti-fatigue material, and showed that it improved physical stamina [7]. These properties and effects might account for the fact that *B. multifida* has been used in Iranian folk medicine to enhance physical strength [10]. In addition, *B. multifida* total root extracts exhibited anxiolytic effect in an elevated plus-maze assay [23]. This finding led to the isolation and characterization of three active coumarin derivatives from *B. multifida* root extracts, namely umbelliferone (**33**), scopoletin (**34**) and ferulic acid (**35**) with the well-known potent monoamine oxidase (MAO) inhibitory and anti-anxiety effects [23,65,66]. These discoveries explain and provide scientific evidence to support the traditional use of *B. multifida* for the management of anxiety.

#### 6.1.4. Hypolipidemic Effect

It has been well-established that lipoproteins play vital roles in atherosclerosis [67,68]. Over the past several decades, a number of studies have indicated that low-density lipoproteins (LDL) and high-density lipoproteins (HDL) have opposite influences as risk factors in the onset and progression of atherosclerosis [67,69,70,71,72,73]. It was verified in the last 20 years that lowering LDL-cholesterol successfully prevents atherosclerosis [74]. *B. multifida* root extracts, prepared by using a solution of water and ethanol with the ratio of 1:2, significantly reduced both the HDL and LDL levels in mice serum at doses of 4 and 5 mg/kg, respectively [75]. In addition, the hydro-methanolic extract of *B. multifida* roots was recently observed to possess a protective effect on ethanol-induced gastric ulcer in rats, which was thought to be partly related to antioxidant activity and accelerating nitric oxide (NO) production in vivo after the rats were treated with the extracts [76]. Taken together, *Biebersteinia* species can be explored for a wide range of pharmacological activities.

### 6.2. In Vitro Pharmacological Activities

#### 6.2.1. Antimicrobial Effects

A number of studies have reported that *B. heterostemon* and *B. multifida* extracts significantly inhibited the growth of various bacteria and fungi in a concentration-dependent manner. For instance, the whole plant extracts of *B. heterostemon* substantially inhibited the growth and proliferation of the pathogenic fungi *Fusarium equiseti*, *F. oxysporum* and *F. moniliforme*, which are thought to be the causes of inducing the Chinese Angelica stem nematode disease, with the minimum inhibitory concentrations (MICs) of 0.6250 mg/mL, 0.6250 mg/mL and 1.2500 mg/mL, respectively [77]. Another independent study estimated the antibacterial activities of root extracts from *B. multifida* against various Gram-positive or negative bacteria, including *Bacillus cereus*, *Clostridium perfringens*, *Staphylococcus aureus*, *Escherichia coli*, *Enterobacter aerogenes* and *Salmonella enterica* [78]. The results unraveled that the root extracts of *B. multifida* obtained by *n*-hexane and ethanol maceration displayed significant antibacterial effects with the MIC of 0.195 mg/mL [78]. Some terpene compounds isolated from *B. heterostemon* were confirmed to possess antibacterial activities. Moreover, the compound (-)-anymol-8-*O*-β-D-lyxopyranoside (**38**), a bisabolane-type sesquiterpene glycoside isolated from *B. heterostemon*, displayed a pronounced antibacterial efficacy against *B. subtilis*, *S. aureus* and *Pseudomonas* spp. with the MICs of 50 μg/mL, 50 μg/mL and 70 μg/mL, respectively [26]. In addition, the prenylated guanidine known as galegine (**30**) was reported to exhibit the most potent antibacterial efficacy against various *S. aureus* strains, including the two methicillin-resistant ones, in the concentration range between 20 and 31 µM [79,80].

#### 6.2.2. Antioxidant Activities

Numerous studies have reported on the antioxidant activities of dietary phenolic substances like flavonoids in various living organisms, including plants, animals and humans [81,82,83,84,85,86]. Most of the published reports have focused on the antioxidant activities of phenolics possessing the ability to inhibit the formation of free radicals, the mode of which depends mainly on the structure-activity relationships of antioxidant compounds [87,88,89,90]. High CTF in *Biebersteinia* plants is closely correlated with their antioxidant activities [17]. Various methods, including 1,1-diphenyl-2-picrylhydrazyl (DPPH) and 2,2′-azinobis(3-ethylbenzothiazoline-6-sulfonic acid) (ABTS) radical-scavenging approaches, and Oil Stability Index (OSI) assay, have been used to determine the antioxidant activities of different *B. heterostemon* solvent extracts, and disclosed that their antioxidant activities varied considerably [91]. Specifically, the ethyl acetate and ethanolic extracts of *B. heterostemon* aerial parts were presented with higher antioxidant activities than the *n*-hexane extract, and their relative efficacies were concentration-dependent [91]. One possible explanation is that flavonoids and phenols were more readily extracted with polar than nonpolar solvents [92,93,94]. In addition, a *B. multifida* root extract was found to be enriched with phenolic compounds (80.1 ± 3.10 mg/mL), and demonstrated strong DPPH radical-scavenging activity (95.9 ± 3.20 μg/mL) [21]. It is worth mentioning that the polyphenolic compounds identified in food products prepared from various plant sources like *Avena sativa*, *Aristotelia chilensis*, *Paeonia ostii* and *Linum usitatissimum* possess significant antioxidant activities as well [95,96,97,98].

Besides antioxidant activities related to phenolic compounds present in *Biebersteinia* spp., the essential oils from different types of tissues of *Biebersteinia* plants were also shown to have strong radical-scavenging activities. For instance, the essential oil of *B. multifida* fruits, evaluated by DPPH assay, was shown to be superior to essential oils extracted from other organs (e.g., leaves and roots), displaying the IC_50_ value of 16.7 ± 0.02 μg/mL that was even more excellent than the well-known synthetic antioxidant butylated hydroxytoluene (BHT, 19.0 ± 0.80 μg/mL) [45]. The chemical composition in the essential oils of *B. multifida* fruits should be responsible for their antioxidant activity due to the antioxidative properties of thymol, 1,8-cineol and β-caryophyllene [45], which are the major compounds in *B. multifida* essential oils [99,100]. These investigations indicate that the *Biebersteinia* species are a valuable natural resource for extracting antioxidant compounds.

#### 6.2.3. Anti-Cancer Effects

A growing body of literature has demonstrated that *Biebersteinia* species possess some other valuable pharmacological effects, in addition to those overviewed in previous subsections. For instance, an ethanolic extract from *B. multifida* roots was reported to prevent mutation reversion by 51.2% in an anti-mutagenicity test, indicating that *B. multifida* plants harbor natural products that can act as anticancer agents [101]. Another independent study showed that the root extract of *B. multifida* obtained by maceration with 70% ethanol was cytotoxic and apoptotic to both human prostate cancer cells DU145 and PC3 in a dose-dependent manner, as this extract significantly decreased cell viability [102].

## 7. Conclusions and Future Perspectives

Natural plant products have been used extensively and widely in traditional medicine, and are important sources for drug discovery and development. Up-to-date, only a few studies have examined and analyzed the phytochemical constituents, bioactivities, and pharmacological aspects and characteristics of *Biebersteinia* species. More than 40 secondary metabolites have been isolated and identified in the members of this plant genus, of which flavonoids were the principal constituents. The varied properties and efficacies of the pharmacologically active substances in different *Biebersteinia* species suggest that these compounds are potential sources of new drugs.

However, certain key issues must be resolved before the identified *Biebersteinia* species can be fully exploited as bases for new pharmaceutical agents. Currently, many of their phytochemical constituents have not yet been systematically identified, and some of those that have already been elucidated do not necessarily account for their observed pharmacological effects. Although certain constituents have significant pharmaceutical effects, their underlying mode-of-action and molecular mechanisms remain unclear. Moreover, in vivo and in vitro models should be designed and implemented in order to screen for unrecognized bioactivities. For instance, although *B. heterostemon* is widely used in folk medicine in northwest China, it has also generally been regarded as toxic, or a weed that is difficult to be eradicated. Consequently, the potential utility of this resource has been underexploited, or even was lost altogether. In order to harness the full value of the identified *Biebersteinia* species as pharmaceutical agents, we should perform basic research on their bioactive constituents, pharmacological properties and molecular mechanisms, which is followed by clinical tests. In-depth investigations are therefore required to develop, test and optimize the administration of novel drugs derived from various organs of plants of this genus. Overall, we believe that this synopsis will facilitate the development and exploitation of new drug resources from these plant materials.

## Figures and Tables

**Table 1:** Chemical constituents (except essential oil-related compounds) in *Biebersteinia* species.

No.	Compound Name	Sources	References
**Flavonoids**			
**1**	luteolin	*B. heterostemon*	[11,13,15]
		*B. multifida*	
		*B. orphanidis*	
**2**	6-hydroxyluteolin	*B. heterostemon*	[13]
**3**	4′-methoxytricetin	*B. heterostemon*	[14]
**4**	5,7,3′-trihydroxy-8,4′,5′-trimethoxyflavone	*B. heterostemon*	[11]
**5**	apigenin	*B. orphanidis*	[15]
**6**	acacetin	*B. orphanidis*	[15]
**7**	5,7,4′-trihydroxy-6,8-dimethoxyflavone	*B. orphanidis*	[15]
**8**	nevadensin	*B. orphanidis*	[15]
**9**	gardenin B	*B. orphanidis*	[15]
**10**	acerosin	*B. orphanidis*	[15]
**11**	sudachitin	*B. orphanidis*	[15]
**12**	hymenoxin	*B. orphanidis*	[15]
**13**	quercetin	*B. heterostemon*	[12]
**14**	myricetin	*B. odora*	[16]
**15**	artemetin	*B. heterostemon*	[23]
**16**	hypolaetin-7-*O*-β-D-xylopyranoside	*B. heterostemon*	[12]
**17**	hypolaetin-7-*O*-β-D-glucopyranoside	*B. heterostemon*	[13]
**18**	3′,4′,5,8-tetrahydroxyflavanone-7-O-β-glucopyranoside	*B. heterostemon*	[24]
**19**	luteolin-7-*O*-glucoside	*B. heterostemon*	[11,13,15]
		*B. multifida*	
		*B. orphanidis*	
**20**	apigenin-7-*O*-glucoside	*B. orphanidis*	[15]
**21**	tricetin-7-*O*-glucoside	*B. orphanidis*	[15]
**22**	diosmin	*B. heterostemon*	[13]
**23**	apigenin-7-*O*-rutinoside	*B. heterostemon*	[13,15]
		*B. orphanidis*	
**24**	luteolin-7-*O*-rutinoside	*B. heterostemon*	[13,15]
		*B. multifida*	
**25**	apigenin-7-*O*-sophoroside	*B. heterostemon*	[13]
**26**	chrysoeriol-7-*O*-sophoroside	*B. heterostemon*	[13]
**27**	quercetin-7-*O*-glucoside	*B. heterostemon*	[11]
**28**	quercetin-3-*O*-β-glucopyranoside	*B. heterostemon*	[13]
**29**	quercetin-3-*O*-β-D-glucopyranosyl	*B. heterostemon*	[13]
	(1→2)-β-D-glucopyranoside		
**Guanidines**			
**30**	galegine	*B. heterostemon*	[12,25]
**31**	*cis*-4-hydroxy galegine	*B. heterostemon*	[25]
**32**	*trans*-4-hydroxy galegine	*B. heterostemon*	[25]
**Phenylpropanoids**			
**33**	umbelliferone	*B. multifida*	[23]
**34**	scopoletin	*B. multifida*	[23]
**35**	ferulic acid	*B. multifida*	[23]
**Terpenoids**			
**36**	geniposide	*B. heterostemon*	[26]
**37**	6β-hydroxy geniposide	*B. heterostemon*	[26]
**38**	(-)-anymol-8-*O*-β-D-lyxopyranoside	*B. heterostemon*	[26]
**Other Types**			
**39**	(+)-dehydrovomifoliol	*B. heterostemon*	[24]
**40**	*N*-3-methyl-2-butenyl urea	*B. heterostemon*	[11]
**41**	vasicinone	*B. multifida*	[27]
**42**	alternariol	*B. heterostemon*	[24]
**43**	mannitol	*B. heterostemon*	[12]
**44**	β-sitosterol	*B. heterostemon*	[11,12,24]
**45**	daucosterol	*B. heterostemon*	[12]
**46**	protocatechuic acid methyl ester	*B. heterostemon*	[24]
**Fatty Acids**			
**47**	myristic acid	*B. orphanidis*	[28]
**48**	palmitic acid	*B. heterostemon*	[28,29]
		*B. orphanidis*	
**49**	stearic acid	*B. heterostemon*	[28,29]
		*B. orphanidis*	
**50**	arachidic acid	*B. heterostemon*	[29]
**51**	docosanoic acid	*B. orphanidis*	[28]
**52**	tetracosanoic acid	*B. orphanidis*	[28]
**53**	hexacosanoic acid	*B. orphanidis*	[28]
**54**	palmitoleic acid	*B. heterostemon*	[28,29]
		*B. orphanidis*	
**55**	oleic acid	*B. heterostemon*	[28,29]
		*B. orphanidis*	
**56**	eicosenoic acid	*B. heterostemon*	[28,29]
		*B. orphanidis*	
**57**	linoleic acid	*B. heterostemon*	[28,29]
		*B. orphanidis*	
**58**	α-linolenic acid	*B. heterostemon*	[28,29]
		*B. orphanidis*	
**59**	γ-linolenic acid	*B. heterostemon*	[29]
**60**	7,10,13-hexadecatrienoic acid	*B. orphanidis*	[28]

**Table 2:** Chemical compositions of essential oils in *Biebersteinia* species.

No.	Compound Name	Molecular Formula	Retention Indices (RI)	Sources	References
1	α-thujene	C_10_H_16_	920	*B. multifida*	[47]
2	α-pinene	C_10_H_16_	939	*B. multifida*	[44,45,46,48]
				*B. heterostemon*	
3	camphene	C_10_H_16_	946	*B. multifida*	[45,48]
				*B. heterostemon*	
4	sabinene	C_10_H_16_	970	*B. multifida*	[45]
5	β-pinene	C_10_H_16_	978	*B. multifida*	[45,48]
				*B. heterostemon*	
6	6-methyl-5-hepten-2-one	C_8_H_14_O	988	*B. multifida*	[47]
7	myrcene	C_10_H_16_	991	*B. multifida*	[45,48]
				*B. heterostemon*	
8	α-phellandrene	C_10_H_16_	1005	*B. multifida*	[45]
9	α-terpinene	C_10_H_16_	1018	*B. multifida*	[45]
10	*p*-cymene	C_10_H_14_	1025	*B. heterostemon*	[48]
11	limonene	C_10_H_16_	1029	*B. multifida*	[45,48]
				*B. heterostemon*	
12	1,8-cineole	C_10_H_18_O	1033	*B. multifida*	[45,47,48]
				*B. heterostemon*	
13	*trans*-β-ocimene	C_10_H_16_	1050	*B. heterostemon*	[48]
14	γ-terpinene	C_10_H_16_	1062	*B. multifida*	[45,48]
				*B. heterostemon*	
15	*trans*-sabinene hydrate	C_10_H_18_O	1064	*B. multifida*	[44,45]
16	linalool	C_10_H_18_O	1099	*B. multifida*	[44,45,48]
				*B. heterostemon*	
17	nonanal	C_9_H_18_O	1102	*B. multifida*	[45]
18	octyl acetate	C_10_H_20_O_2_	1124	*B. multifida*	[45]
19	*cis*-limonene oxide	C_10_H_16_O	1131	*B. orphanidis*	[43]
20	*trans*-pinocarveol	C_10_H_16_O	1140	*B. multifida*	[45]
21	camphor	C_10_H_16_O	1143	*B. multifida*	[44,47,48]
				*B. heterostemon*	
22	(*Z*)-3-nonenol	C_9_H_18_O	1158	*B. multifida*	[47]
23	pinocarvone	C_10_H_14_O	1164	*B. multifida*	[45]
24	borneol	C_10_H_18_O	1168	*B. multifida*	[47]
25	terpinen-4-ol	C_10_H_18_O	1177	*B. multifida*	[45]
26	α-terpineol	C_10_H_18_O	1189	*B. multifida*	[43,45,47]
				*B. orphanidis*	
27	myrtenal	C_10_H_14_O	1197	*B. multifida*	[45]
28	decanal	C_10_H_20_O	1204	*B. multifida*	[47]
29	*trans*-carveol	C_10_H_16_O	1217	*B. multifida*	[45]
30	carvone	C_10_H_14_O	1242	*B. multifida*	[45,47,48]
				*B. heterostemon*	
31	geraniol	C_10_H_18_O	1255	*B. heterostemon*	[48]
32	linalyl acetate	C_12_H_20_O_2_	1257	*B. orphanidis*	[43]
33	isobornyl acetate	C_12_H_20_O_2_	1283	*B. multifida*	[47]
34	bornyl acetate	C_12_H_20_O_2_	1285	*B. multifida*	[45]
35	thymol	C_10_H_14_O	1290	*B. multifida*	[45]
36	(2*E*,4*Z*)-decadienal	C_10_H_16_O	1293	*B. multifida*	[47]
37	carvacrol	C_10_H_14_O	1302	*B. multifida*	[47]
38	(2*E*,4*E*)-decadienal	C_10_H_16_O	1316	*B. multifida*	[47]
39	δ-elemene	C_15_H_24_	1339	*B. heterostemon*	[48]
40	α-longipinene	C_15_H_24_	1352	*B. heterostemon*	[48]
41	eugenol	C_10_H_12_O_2_	1361	*B. multifida*	[47]
42	α-ylangene	C_15_H_24_	1374	*B. multifida*	[47]
43	geranyl acetate	C_12_H_20_O_2_	1381	*B. heterostemon*	[48]
44	β-elemene	C_15_H_24_	1391	*B. multifida*	[43,45,48]
				*B. orphanidis*	
				*B. heterostemon*	
45	tetradecane	C_14_H_30_	1400	*B. multifida*	[46]
46	isocaryophyllene	C_15_H_24_	1408	*B. multifida*	[47]
47	*cis*-caryophyllene	C_15_H_24_	1409	*B. heterostemon*	[48]
48	α-gurjunene	C_15_H_24_	1412	*B. orphanidis*	[43]
49	β-caryophyllene	C_15_H_24_	1416	*B. multifida*	[43,44,45,47,48]
				*B. orphanidis*	
				*B. heterostemon*	
50	α-bergamotene	C_15_H_24_	1418	*B. heterostemon*	[48]
51	β-duprezianene	C_15_H_24_	1424	*B. multifida*	[47]
52	γ-elemene	C_15_H_24_	1431	*B. multifida*	[45,48]
				*B. heterostemon*	
53	α-humulene	C_15_H_24_	1449	*B. multifida*	[45,48]
				*B. heterostemon*	
54	β-farnesene	C_15_H_24_	1457	*B. multifida*	[44,45,47,48]
				*B. heterostemon*	
55	allo-aromadendrene	C_15_H_24_	1462	*B. multifida*	[44,48]
				*B. heterostemon*	
56	α-amorphene	C_15_H_24_	1480	*B. heterostemon*	[48]
57	germacrene D	C_15_H_24_	1485	*B. multifida*	[45,48]
				*B. heterostemon*	
58	(*E*)-β-ionone	C_13_H_20_O	1486	*B. multifida*	[47]
59	*cis*-β-guaiene	C_15_H_24_	1487	*B. heterostemon*	[48]
60	*epi*-cubebol	C_15_H_26_O	1495	*B. multifida*	[47]
61	bicyclogermacrene	C_15_H_24_	1495	*B. multifida*	[45]
62	α-selinene	C_15_H_24_	1498	*B. heterostemon*	[48]
63	germacrene A	C_15_H_24_	1501	*B. heterostemon*	[48]
64	β-bisabolene	C_15_H_24_	1505	*B. multifida*	[47]
65	α-farnesene	C_15_H_24_	1507	*B. multifida*	[44]
66	γ-cadinene	C_15_H_24_	1512	*B. multifida*	[44,47]
67	δ-cadinene	C_15_H_24_	1522	*B. multifida*	[44,45,47]
68	d-cadinene	C_15_H_24_	1525	*B. heterostemon*	[48]
69	guaia-3,9-diene	C_15_H_24_	1534	*B. heterostemon*	[48]
70	α-cadinene	C_15_H_24_	1536	*B. multifida*	[44,48]
				*B. heterostemon*	
71	nerolidol	C_15_H_26_O	1538	*B. multifida*	[45,48]
				*B. heterostemon*	
72	eudesma-3,7(11)-diene	C_15_H_24_	1545	*B. heterostemon*	[48]
73	elemol	C_15_H_26_O	1552	*B. multifida*	[44]
74	elixene	C_15_H_24_	1559	*B. heterostemon*	[48]
75	germacrane B	C_15_H_24_	1563	*B. orphanidis*	[43]
76	(*E*)-nerolidol	C_15_H_26_O	1565	*B. multifida*	[44,45,46,47]
77	spathulenol	C_15_H_24_O	1578	*B. multifida*	[43,45,47]
				*B. orphanidis*	
78	caryophyllene oxide	C_15_H_24_O	1583	*B. multifida*	[43,44,45,47,48]
				*B. orphanidis*	
				*B. heterostemon*	
79	viridiflorol	C_15_H_26_O	1594	*B. multifida*	[44]
80	hexadecane	C16H34	1600	*B. multifida*	[46]
81	guaiol	C_15_H_26_O	1601	*B. multifida*	[47]
82	humulene epoxide II	C_15_H_24_O	1610	*B. multifida*	[44]
83	β-elemenone	C_15_H_22_O	1612	*B. heterostemon*	[48]
84	dillapiole	C_12_H_14_O_4_	1624	*B. multifida*	[47]
85	τ-cadinol	C_15_H_26_O	1635	*B. multifida*	[44,45]
86	*epi*-α-cadinol	C_15_H_26_O	1642	*B. multifida*	[43,47]
				*B. orphanidis*	
87	α-eudesmol	C_15_H_26_O	1656	*B. multifida*	[44,47]
88	α-bisabolol oxide B	C_15_H_26_O_2_	1658	*B. orphanidis*	[43]
89	bulnesol	C_15_H_26_O	1671	*B. multifida*	[44,47]
90	(*Z*)-α-santalol	C_15_H_24_O	1674	*B. orphanidis*	[43]
91	*cis*-β-elemenone	C_15_H_22_O	1678	*B. heterostemon*	[48]
92	α-bisabolol	C_15_H_26_O	1688	*B. multifida*	[43,44,47,48]
				*B. orphanidis*	
				*B. heterostemon*	
92	germacrone	C_15_H_22_O	1699	*B. heterostemon*	[48]
94	(*E*)-nerolidol acetate	C_15_H_26_O	1714	*B. multifida*	[44,47]
95	(2*E*,6*E*)-farnesol	C_15_H_26_O	1727	*B. multifida*	[44,47]
96	octadecane	C_18_H_38_	1800	*B. multifida*	[46,47]
97	neophytadiene	C_20_H_38_	1836	*B. multifida*	[46]
98	6,10,14-trimethyl-2-pentadecanone	C_18_H_36_O	1845	*B. multifida*	[44,46,47]
99	nonadecane	C_19_H_40_	1900	*B. multifida*	[47]
100	farnesyl acetone	C_18_H_30_O	1917	*B. multifida*	[44,47]
101	methyl linolenate	C_19_H_32_O_2_	2098	*B. multifida*	[47]
102	phytol	C_20_H_40_O	2124	*B. multifida*	[44,47]
104	mandenol	C_20_H_36_O_2_	2148	*B. multifida*	[44]
104	ethyl linolenate	C_20_H_34_O_2_	2162	*B. multifida*	[44,47]
105	10-cyclohexyl-nonadecane	C_25_H_52_	2312	*B. multifida*	[44]
106	pentacosane	C_25_H_52_	2517	*B. multifida*	[44]
107	*n*-heptacosane	C_27_H_56_	2682	*B. multifida*	[44]
108	octacosane	C_28_H_58_	2791	*B. multifida*	[44]
109	nonacosane	C_29_H_60_	2894	*B. multifida*	[44]
110	vitamin E	C_29_H_50_O_2_	3138	*B. multifida*	[44]
111	*n*-docosane	C_22_H_46_	—	*B. multifida*	[44]
112	epizonaren	C_15_H_24_	—	*B. multifida*	[44]
